# Reliable smart models for estimating frictional pressure drop in two-phase condensation through smooth channels of varying sizes

**DOI:** 10.1038/s41598-024-60898-7

**Published:** 2024-05-07

**Authors:** M. A. Moradkhani, S. H. Hosseini, Mengjie Song, A. Abbaszadeh

**Affiliations:** 1 Department of Chemical Engineering, Ilam University, Ilam, 69315-516 Iran; 2 Department of Energy and Power Engineering, School of Mechanical Engineering, Beijing Institute of Technology, Beijing, 100081 China; 3 School of Mechanical Engineering, Hanyang University, Seoul, 04763 South Korea; 4 Department of Civill Engineering, Ilam University, Ilam, 69315-516 Iran

**Keywords:** Frictional pressure drop, Condensation, Machine learning algorithms, Modeling, Mini/micro and conventional channels, Chemical engineering, Mechanical engineering

## Abstract

Reliable and comprehensive predictive tools for the frictional pressure drop (FPD) are of particular importance for systems involving two-phase flow condensation. However, the available models are only applicable to specific operating conditions and channel sizes. Thus, this study aims at developing universal models to estimate the FPD during condensation inside smooth mini/micro and conventional (macro) channels. An extensive databank, comprising 8037 experimental samples and 23 working fluids from 50 reliable sources, was prepared to achieve this target. A comprehensive investigation on the literature models reflected the fact that all of them are associated with high deviations, and their average absolute relative errors (AAREs) exceed 26%. Hence, after identifying the most effective input variables through the Spearman's correlation analysis, three soft-computing paradigms, i.e., multilayer perceptron (MLP), gaussian process regression (GPR) and radial basis function (RBF) were employed to establish intelligent and dimensionless predictive tools for the FPD based on the separated model suggested by Lockhart and Martinelli. Among them, the most accurate results were presented by the GPR approach with AARE and $R^{2}$ values of 4.10%, 99.23% respectively, in the testing step. The truthfulness and applicability of the models were explored through an array of statistical and visual analyses, and the results affirmed the obvious superiority of the newly proposed approaches over the literature correlations. Furthermore, the novel predictive tools excellently described the physical variations of the condensation FPD versus the operating parameters. Ultimately, the order of importance of factors in controlling the condensation FPD was clarified by a sensitivity analysis.

## Introduction

Heat exchangers involving two-phase flow condensation are widely utilized in numerous industries, such as nuclear, food processing, refrigeration, air conditioning, etc^1–6^. During the last years, compact heat exchangers (mini/micro channels) have attracted special attention, since they provide much higher energy efficiency, require lower amounts of refrigerant, and take up less space compared to conventional ones^7–9^. However, the enhanced flow area per mass velocity in these channels leads to higher pressure drop, which is followed by several negative impacts on the system^10^. Increasing the energy consumption in pumps and obstructing the two-phase flow system are well-known examples regarding the destructive influences of high pressure drop inside channels^11–13^. Consequently, the optimal design of heat exchangers necessitates comprehensive predictive tools for pressure drop in channels of various sizes.

The total pressure drop during two-phase flow inside channels is defined as the sum of three different terms, including frictional, gravitational and accelerational pressure drops,

1 $$\left( {\frac{dP}{{dz}}} \right)_{tp,T} = \left( {\frac{dP}{{dz}}} \right)_{tp,F} + \left( {\frac{dP}{{dz}}} \right)_{tp,G} + \left( {\frac{dP}{{dz}}} \right)_{tp,A}$$

The gravitational and acceleration terms are given by,

2 $$\left( {\frac{dP}{{dz}}} \right)_{tp,A} = G^{2} \frac{d}{dz}\left[ {\frac{{\left( {1 - x} \right)^{2} }}{{\rho_{l} \left( {1 - \alpha } \right)}} + \frac{{x^{2} }}{{\rho_{v} \alpha }}} \right]$$

3 $$\left( {\frac{dP}{{dz}}} \right)_{tp,G} = g\left[ {\rho_{l} \left( {1 - \alpha } \right) + \rho_{v} \alpha } \right]\sin \left( \omega \right)$$

where the void fraction, $\alpha$ is calculated by Eq. (4) proposed by Zivi^14^.

4 $$\alpha = \left[ {\left( {\frac{{\rho_{v} }}{{\rho_{l} }}} \right)^{\frac{2}{3}} \left( {\frac{1 - x}{x}} \right) + 1} \right]^{ - 1}$$

According to earlier experimental investigations, the frictional pressure drop (FPD) envelopes more than 90% of total pressure drop^15–18^. Thereupon, it is vital to derive precise and reliable approaches for estimating the two-phase FPD during condensation, covering both mini/micro and macro channels.

Several theoretical and empirical correlations can be found in the literature for predicting the FPD during condensation inside channels^19–29^. Most of these correlations have been established by inspiring the homogeneous and separated models. The homogenous approach supposes the flow as a pseudo single-phase flow, in which both phases have the same velocity. However, the earlier studies have implied that this method is not reliable for low pressures and mass fluxes^30,31^. In the separated model presented by Lockhart and Martinelli^32^, FPD is determined by modification of the single-phase pressure drop by applying a two-phase multiplier,

5 $$\left( {\frac{dP}{{dz}}} \right)_{tp,F} = \left( {\frac{dP}{{dz}}} \right)_{vo} \phi_{vo}^{2} = \left( {\frac{dP}{{dz}}} \right)_{lo} \phi_{lo}^{2} = \left( {\frac{dP}{{dz}}} \right)_{v} \phi_{v}^{2} = \left( {\frac{dP}{{dz}}} \right)_{l} \phi_{l}^{2}$$

where the pressure drop of various phases are defined as follows,

6a $$\left( {\frac{dp}{{dz}}} \right)_{l} = \frac{{2\left( {1 - x} \right)^{2} G^{2} f_{l} }}{{D\rho_{l} }}$$

6b $$\left( {\frac{dp}{{dz}}} \right)_{v} = \frac{{2x^{2} G^{2} f_{v} }}{{D\rho_{v} }}$$

6c $$\left( {\frac{dp}{{dz}}} \right)_{lo} = \frac{{2G^{2} f_{lo} }}{{D\rho_{l} }}$$

6d $$\left( {\frac{dp}{{dz}}} \right)_{vo} = \frac{{2G^{2} f_{vo} }}{{D\rho_{v} }}$$

where the friction factor,$f$ for a given phase (*k*) can be calculated by,

7a $$f_{k} = \frac{16}{{Re_{k} }}\;for\;Re_{k} < 2000$$

7b $$f_{k} = \frac{0.079}{{Re_{k}^{0.25} }}\;for\;2000 \le Re_{k} < 20,000$$

7c $$f_{k} = \frac{0.046}{{Re_{k}^{0.2} }}\;for\;Re_{k} \ge 20,000$$

According to Chisholm^33^ theory, the two-phase multiplier is defined as function of Lockhart and Martinelli parameter, i.e., *X*,

8 $$\phi_{l}^{2} = \frac{1}{{X^{2} }} + \frac{C}{X} + 1$$

9 $$X = \sqrt {{\raise0.7ex\hbox{${\left( {\frac{dp}{{dz}}} \right)_{l} }$} \!\mathord{\left/ {\vphantom {{\left( {\frac{dp}{{dz}}} \right)_{l} } {\left( {\frac{dp}{{dz}}} \right)_{v} }}}\right.\kern-0pt} \!\lower0.7ex\hbox{${\left( {\frac{dp}{{dz}}} \right)_{v} }$}}}$$

According to this methodology, the Chisholm parameter, C may experience alternations from 5 to 20, depending on the flow regime. Sun and Mishima^34^ developed a correlation for FPD in mini/micro channels based on Chisholm method, in which $Re_{l}$ and $Re_{v} = 2000$ were defined as the transition point for Chisholm parameter. An AARE value of 30.6% for all analyzed data was yielded by this model. Hossain et al^35^. utilized their own experimental data for correlating the Chisholm parameter with Froud and Bond Numbers. The reasonable consistencies between measured data and those predicted by the correlation were testified with AARE of 9.51%. Jige et al^36^. estimated the two-phase multiplier using their experimental data for condensation of four different refrigerants in multiport mini-channels, and obtained a reasonable accuracy with AARE of 9.6%.

Various FPD models have also been proposed based on extensive sets of experimental data. However, most of these models are applicable for specific channel sizes. The Friedel^37^ model was developed by employing 25,000 data for macro channels with diameters higher than 4 mm and working fluids of air-oil, R12, and air–water. Another comprehensive model was suggested by Muller-Steinhagen an Heck^38^ utilizing around 9300 data points for channel diameters exceeding 4 mm. Although the database encompassed various types of working fluids, the number of data corresponding to air–water was remarkably larger than the rests. Kim and Mudawar^39^ proposed an universal predictive tool for condensation FPD in mini/micro channels with diameters up to 6.25 mm. In order to attain more accurate predictions, the analyzed data were allocated to four subdomains, depending on the vapor and liquid Reynolds numbers. A total number of 7115 data points analyzed for the condensation FPD were predicted with an AARE of 23.3% by this model. There is just one correlation in the literature applicable for both mini/micro and conventional channels, which has been presented by Moradkhani et al^40^. This correlation was derived by implementing the genetic programming approach over a widespread databank, containing 7328 FPD samples, and exhibited satisfactory outcomes for different sizes of channels.

Recently, intelligent techniques, such as machine learning algorithms have been broadly employed to solve the engineering problems ^41–48^. There some limited studies on the application of the foregoing approaches to model the two-phase pressure drop in heat exchangers of various configuration. Zendehboudi and Li^49^ studied the application of four intelligent methods in estimating the condensation FPD in inclined tubes based on 312 data from just one source. All established models provided reliable estimations with $R^{2}$ values exceeding 95% for the test data. López-belchí et al^50^. employed the group method of data handling (GMDH) approach to estimate the condensation FPD inside micro-channels based on their own measured data, and yielded a total AARE of 10.59%. Longo et al^51^. developed a predictive method for two-phase pressure drop in plate heat exchangers through the gradient boosting machines (GBM). The modeling phase was performed by utilizing 925 data for flow condensation and 1624 data for flow boiling. It was demonstrated that the intelligent approaches perform much better than the empirical correlations. In another relevant work, Qiu et al^13^. suggested several intelligent models for boiling FPD in mini/micro channels using 2787 data from 21 studies. Among them, the MLP based model, which included 12 hidden layers and 23 dimensionless groups as inputs provided the superior outcomes. Moradkhani et al^52^. assessed some neural network-based approaches for estimating the two-phase FPD inside helically coiled tubes. Their analyzed dataset included 1267 experimental samples adopted from 12 studies. The most precise outcomes were obtained by the radial basis function (RBF) method with AARE of 4.73% during the testing phase. More recently, Montañez-barrera et al^53^. studied the application of correlated-informed neural networks (CoINN) for modeling the FPD of zeotropic mixtures during two-phase flow in micro-channels. The model overcame the available correlations with a total AARE of 6% from the actual data.

This study addresses critical limitations in two-phase FPD estimation during condensation. Existing correlations are often limited to specific channel sizes, and the predictive tools applicable to both mini/micro and conventional channels are rare. On the other hand, the machine learning approaches haven't been explored for developing generalizable models for condensation FPD inside smooth channels of various sizes. Furthermore, identifying the key factors influencing FPD is crucial for engineers. To address these gaps, this study analyzes a comprehensive dataset of 8037 samples encompassing mini/micro and conventional channels from 50 published studies. For the first time, three important soft computing approaches, namely, MLP, GPR and RBF are implemented to establish novel models for FPD estimation. The correctness and validity of the proposed models are statistically assessed for estimating the FPD in various channel sizes, flow patterns, and flow regimes. Furthermore, the outputs of the models are utilized to examine the changes in condensation FPD with respect to different operational factors. A sensitivity analysis is then used to identify the most effective factors on FPD.

## Materials and methods

### Machine learning algorithms

To address the need for robust predictive tools in two-phase FPD estimation during condensation in channels, this study employed three widely recognized machine learning algorithms: MLP, GPR, and RBF. These approaches were chosen due to their success in describing complex two-phase flow behavior, as demonstrated in previous studies^52,54–56^.

#### MLP

**Figure 1:**
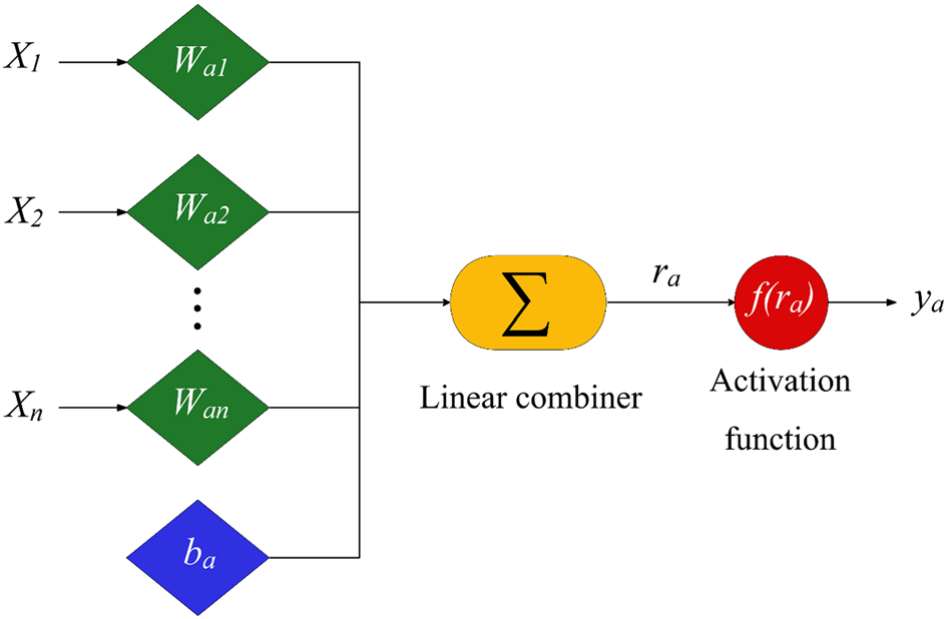
Description of an artificial neuron.

The capable machine learning approach of MLP follows a process similar to that observed in the nervous system of humans, and it is mainly implemented to solve the complicated mathematical problems, including approximation, classification and pattern recognition^57^. This is done through a parallel algorithm, in which a set of data is utilized to train the network, and the artificial neurons are responsible for transferring the information^58^. Figure 1 shows the structure of an artificial neuron included in the MLP network. Mathematically, this neuron is explained as follow,

10 $$r_{a} = \mathop \sum \limits_{i = 1}^{n} x_{i} W_{ai} + b_{a}$$

11 $$y_{a} = f\left( {r_{a} } \right)$$

where $r_{a}$, $x_{i}$, $W_{ai}$, $b_{a}$ and stand for linear combiner, *i*th input factor, synaptic weight, neuron bias and activation function, respectively.

**Figure 2:**
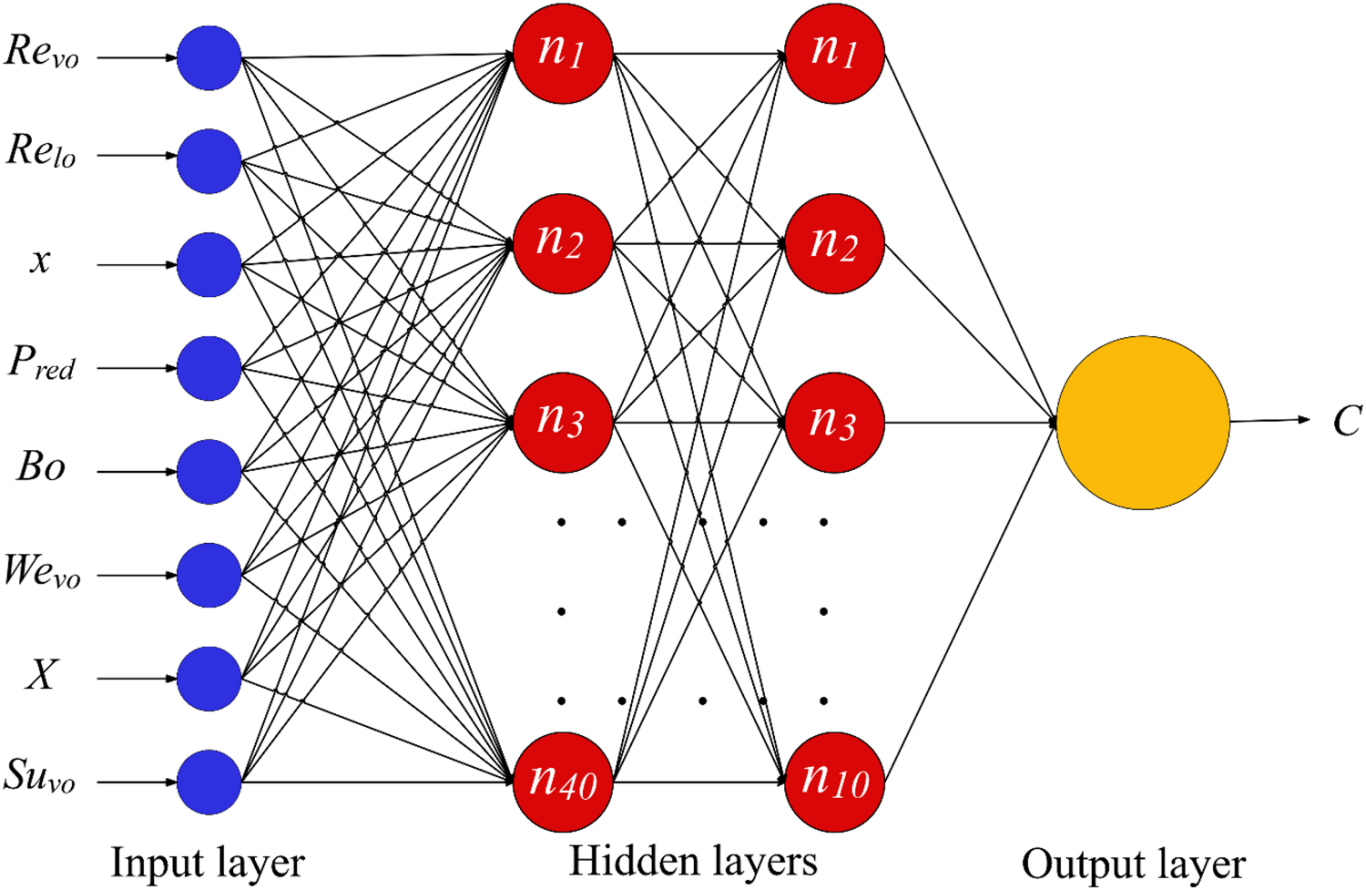
The MLP network designed to estimate the condensation FPD.

MLP is recognized as feed-forward network, meaning it processes the information only in one direction. The graphical description regarding the MLP network designed to model the condensation FPD has been illustrated in Fig. 2. It is clear that it entails three linked layers, including some artificial neurons. The input, hidden and output layers are responsible for introducing the information to the network, specify network parameters and display the outcomes, respectively. It should be emphasized that the architecture of hidden layer depends on the complexity of problems, and may include some independent layers with different numbers of neurons in each of them. While, the number of neurons in the first and last layers equal to the number of input and output variables, respectively. The MLP network detects the system nonlinearity via the activation functions included in the hidden layer's neurons.

This study identified a four-hidden layer MLP network with (40, 30, 20, 10) neurons per layer as the optimal architecture for modeling condensation FPD. The number of neurons in input and output layers are 8 and 1, respectively. Moreover, the neurons benefited from the tan-sigmoid function as activation function^59^.

A vital stage in designing the MLP network is optimizing its weights and biases, as these parameters significantly influence the network's performance. For this purpose, the back propagation (BP) algorithm specifies the weights and biases to minimize a deviation function^60^,

12 $$D = \left( {y_{pre} - y_{\exp } } \right)^{2}$$

After applying each training data, the value of deviation function is spread in the network, followed by re-adjusting the parameters. Herein, the Bayesian regularization approach was used to train the BP algorithm.

#### GPR

Recently, due to robustness and capabilities of non-parametric machine learning approaches, trends toward them in engineering application have been increased. Among the foregoing approaches, GPR is a widely used machine learning algorithm, by which a gaussian joint probability distribution is provided^61,62^. The main preponderances of GPR are high accuracy and strength to modulate the hyper-parameters. This method is also capable to catch the uncertainty of analyzed samples.

The GPR-based learning process is accomplished through a probabilistic framework, in which a training dataset is provided. It should be noted that and stand for the input variables vector and target function, respectively. Thus, the predictive model provides the output function distribution in any point through the following approximation^54^,

13 $$y_{i} = L\left( {x_{i} } \right) + \varepsilon_{i}$$

where $L\left( {x_{i} } \right)$ is the latent function corresponding to the input variables ($x_{i}$), and its values constitutes a random variable. Furthermore, $\varepsilon_{i}$ represents the gaussian noise, which its mean and variance are 0 and $\sigma_{n}^{2}$, respectively^63^,

14 $$\varepsilon_{i} = N\left( {0,\sigma_{n}^{2} } \right)$$

Consequently, the target function can be easily approximated by defining a mean function $m\left( x \right)$ and a covariance function $cov\left( {x,x^{\prime}} \right)$. The predictive probability distribution for the input variables, $x^{*}$ can be defined as,

15 $$\hat{y}^{*} = m\left( {x^{*} } \right) + k_{*}^{T} \left( {K + \sigma_{n}^{2} I} \right)^{ - 1} \left( {y - m\left( {x^{*} } \right)} \right)$$

16 $$\sigma_{{y^{*} }}^{2} = k_{*} + \sigma_{n}^{2} - k_{*}^{T} \left( {K + \sigma_{n}^{2} I} \right)^{ - 1} k_{*}$$

where $k_{*}$ may be defined as $\left[ {k_{*} } \right]_{i} = cov\left( {x_{i} ,x^{*} } \right)$, $K$ stands for a covariance matrix, which its elements are $\left[ K \right]_{i,j} = cov\left( {x_{i} ,x_{j} } \right)$, and $I$ shows the identity matrix.

As the predictive probability distribution is specified by the hyperparameters, an optimization process should be performed in order to determine these factors^64,65^. In the GPR approach, the log-likelihood function is maximized during the training step in order to calculate the hyperparameters,

17 $$\log p\left( {y{|}X} \right) = - \frac{1}{2}y^{T} \left( {K + \sigma_{n}^{2} I} \right)^{ - 1} y - \frac{1}{2}\log \left( {\left| {\left( {K + \sigma_{n}^{2} I} \right)} \right|} \right) - \frac{n}{2}{\text{log}}\left( {2\pi } \right)$$

where n represents the number of training data points.

#### RBF

Resolving the drawbacks of MLP networks, such as their empirical structure and under or over-fitting possibility is the motivation behind the development of RBF networks. The high potency for interpolation, prompt convergence, simple structure and sublime reliability are the main advantages of these networks^66^. The structure of this network is similar to that of a single-layer MLP network, which includes a number of neurons equal to the number of data points used for training. It should be noted that, the activation functions employed in these networks are radial basis functions, among which the most well-known function is gaussian,

18 $$H\left( x \right) = \exp \left( { - \frac{{\left( {x - c} \right)^{2} }}{{r^{2} }}} \right)$$

where and denote the center and radius of the gaussian function, respectively.

After receiving the information from the input layer, the hidden layer exert the nonlinear functions and sends the results to the final layer, which is responsible to combine the weights and the activation functions, linearly^67,68^,

19 $$f\left( x \right) = \mathop \sum \limits_{i = 1}^{n} W_{i} H_{i} \left( x \right)$$

The gradient descent method is employed in the RBF network to optimize the network center and synaptic weights, minimizing the mean squared error (MSE),

20 $$MSE = \frac{1}{n}\mathop \sum \limits_{i = 1}^{n} \left( {y_{pre} - y_{exp} } \right)^{2}$$

The RBF network used in this study has an 8-6430-1 architecture, signifying 8 neurons in the input layer, 6430 neurons in the hidden layer, and 1 neuron in the output layer. Additionally, the hidden layer employs a Gaussian function as its activation function.

### Experimental data gathering

Since the availability of credible data plays a major role in designing the data-driven models, the collection of as much data as possible regarding the condensation FPD inside channels built the primary cornerstone of the present communication. Hence, a comprehensive dataset, encompassing 8037 FPD samples was adopted from 50 published studies. The foregoing data envelop the FPD of 23 fluids, such as chemicals, halocarbons, natural refrigerants, water, hydrocarbons, cryogens, etc., condensing inside both mini/micro and conventional channels. A detailed description of the operating conditions of the analyzed sources has been provided in Table 1. It should be expressed that the REFPROP v.9.1 software^69^ (https://www.nist.gov/programs-projects/reference-fluid-thermodynamic-and-transport-properties-database-refprop) has been employed to determine the thermophysical characteristics of the fluids under saturation condition.

**Table 1:** Geometrical and operating conditions of analyzed sources for condensation FPD.

Sources	Fluid	Channel geometry	Hydraulic diameter (mm)	Reduced pressure (−)	Mass flux(k)	Number of points
^70^	R717, R744, R245fa	Circular	1.02	0.07–0.69	100–440	238
^71^	R410A	Circular	0.76–3.05	0.8–0.9	200–800	291
^72^	R134a	Circular	8.1	0.25–0.32	300–500	40
^73,74^	R32, R245fa	Circular	0.96	0.07–0.43	200–1000	63
^75,76^	R134a, R1234yf	Circular	2.14	0.14–0.23	50–200	61
^77^	R134a, R1234yf	Square	1.16	0.25–0.49	470–710	81
^78^	R32, R410A	Square	1.16	0.33–0.63	350–800	250
^79^	R290	Square	1.16	0.25–0.40	175–350	109
^80^	R125, R22, R32, R134a, R236ea, R410A	Circular	8	0.1–0.56	100–750	151
^81,82^	R245fa	Circular	3	0.13–0.53	100–1000	174
^83,84^	R134a	Circular, Rectangular, Triangular, Square, Barrel	0.424–1.524	0.30	150–750	561
^85^	R1234yf, R134a	Circular	0.96–2	0.17–0.32	200–800	286
^86^	R290, R600a	Circular	1	0.10–0.22	240–480	147
^87^	R744	Rectangular	0.1–0.16	0.69–0.87	400–800	175
^88^	R1234yf	Circular	3.2–8	0.17–0.23	200–400	162
^89,90^	R744	Circular	1.5	0.41–0.54	400–1000	95
^36^	R32, R1234ze(E), R134a, R410A	Circular	0.85–1.1	0.35–0.78	100–500	284
^91^	R744	Circular	5.15	0.36–0.54	600–1000	52
^92^	R404A	Circular	0.508–3.05	0.38–0.77	200–800	392
^93^	R410A	Circular	6.3	0.56	100–250	12
^15–18^	R152a, R290, R1234ze(E), R22,	Circular, Square	0.952–1.152	0.2–0.4	200–800	293
^94–96^	R290, R1270, R404A, R32, R410A, R1234yf, R1234ze(E), R134a, R152a,	Circular	4	0.15–0.49	75–800	707
^97,98^	R290	Circular	7.75–14.45	0.25–0.95	150–450	276
^99,100^	R601, R245fa	Circular	7.75–14.45	0.03–0.17	100–600	274
^101,102^	R410A	Circular	6.2–9.4	0.80–0.90	200–800	450
^103^	R718	Circular	19	0.18–0.45	400–1000	113
^104^	R134a, R410A	Circular	1.02–1.54	0.10–0.30	50–300	407
^105^	R22, R290, R32, R410A	Rectangular	0.83	0.37–0.60	50–500	112
^106^	R728	Circular	1–2	0.31	32.7–262	58
^107^	R134a	Circular	3.8297–8.91	0.22–0.29	450–650	113
^108,109^	R14, R170	Circular	4	0.27–0.8	200–650	155
^110^	R1234yf, R1234ze(E), R134a, R600a	Square, Circular, Triangular	0.835–1.1	0.11–0.31	100–1400	746
^111^	R1234ze(E), R134a	Circular	1.88	0.17–0.22	450–900	281
^112^	R290	Circular	1.22	0.09–0.14	40–90	30
^113,114^	R50, R170	Circular	4	0.21–0.65	99–251	398
**Total**			0.1–19	0.03–0.95	32.7–1400	**8037**

### Error analysis

In this study, the correctness of various models for estimating the condensation FPD was assayed based on three statistical metrics, including average absolute relative error (AARE), standard deviation (SD), and coefficient of determination,

21 $$AARE \left( \% \right) = \frac{1}{N}\sum \left| {\frac{{FPD_{exp} - FPD_{pre} }}{{FPD_{exp} }}} \right| \times 100$$

22 $$SD \left( \% \right) = \sqrt {\frac{{\sum \left( {E_{i} - \overline{{E_{i} }} } \right)^{2} }}{N - 1}} \times 100$$

23 $$R^{2} \left( \% \right) = \left( {1 - \frac{{\sum \left( {FPD_{exp} - FPD_{pre} } \right)^{2} }}{{\sum \left( {FPD_{exp} - \overline{{FPD_{exp} }} } \right)^{2} }}} \right) \times 100$$

where $E_{i}$ is relative error that can be defined as the following equation,

24 $$E_{i} = \frac{{FPD_{exp} - FPD_{pre} }}{{FPD_{exp} }}$$

## Results and discussion

### Accuracy of literature models

As discussed earlier, several predictive tools can be found in the open literature concerning the estimation of condensation FPD inside channels. A statistical investigation on the accuracy of eleven well-known FPD models based on the collected databank has been presented in Table 2. It is clear that the highest precision among these models belongs to the correlation suggested by Moradkhani et al^40^. with AARE, SD and $R^{2}$ values of 26.23%, 40.23% and 89.05%, respectively from the experimental data. Since the foregoing model has been established based on a widespread FPD dataset enveloping a broad range of conditions in both conventional and mini/micro channels, it is reasonable to provide the highest level of precision. The models presented by Muller-Steinhagen and Heck^38^, Jige et al^36^., Kim and Mudawar^39^, and Sun and Mishima^34^ present almost same results with AAREs of 30.10%, 31.75%, 31.85% and 32.31%, respectively. Such deviations stem from the fact that the mentioned correlations have been recommended for limited geometrical or operating conditions. Nevertheless, their estimation capabilities are superior to those of Friedel^37^, Gu et al^115^., Hossain et al^35^., Zhang et al^116^. and Koyama et al^117^. models, which show AARE values between 34.85 and 61.74%. The weakest performance in predicting the condensation FPD can be attributed to correlation presented by Chisholm^33^ with AARE and SD values of 95.28% and 114.65%, respectively. Overall, the current analysis testifies the requirement for designing comprehensive and precise models to estimate the two-phase FPD during flow condensation in mini/micro and conventional channels.

**Table 2:** Assessment of the literature FPD models based on the collected experimental databank.

Models	AARE, (%)	SD, (%)	$R^{2} ,$(%)
Chisholm^33^	95.28	114.65	84.62
Hossain et al^35^.	51.18	26.49	31.36
Sun and Mishima^34^	32.31	35.19	67.11
Zhang et al^116^.	60.88	91.15	42.36
Koyama et al^117^.	61.74	91.17	52.17
Muller-Steinhagen and Heck^38^	30.10	38.48	59.42
Jige et al^36^.	31.75	43.68	59.33
Friedel^37^	34.85	50.42	58.10
Kim and Mudawar^39^	31.85	41.42	52.17
Gu et al^115^.	38.15	44.19	43.28
Moradkhani et al^40^.	26.23	40.23	89.05

### The novel predictive tools for condensation FPD

#### Optimization of the input variables defined in the models

**Figure 3:**
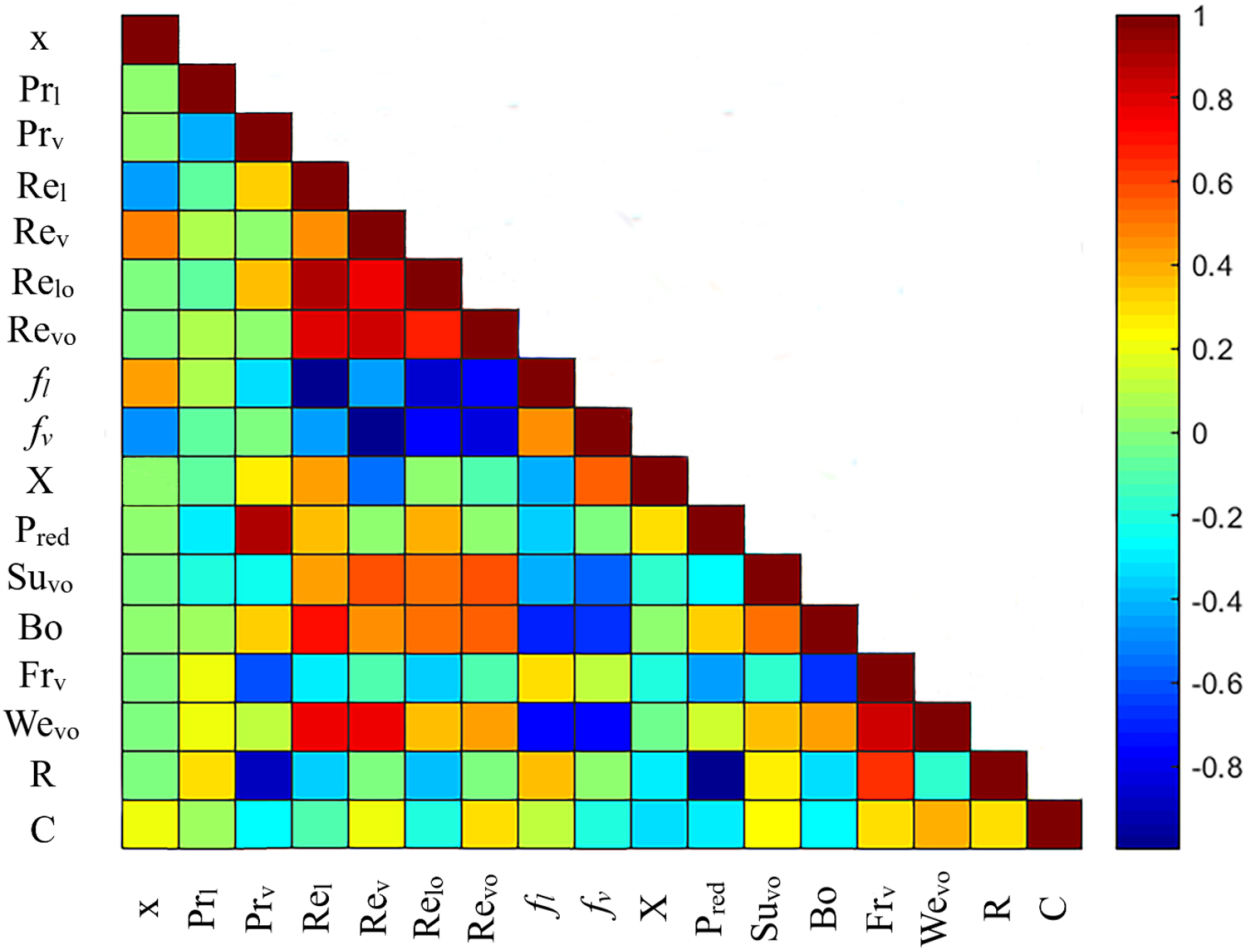
The Spearman's correlation coefficient between different dimensionless groups and Chisholm parameter.

This study introduces novel predictive approaches for estimating condensation FPD based on the separated model proposed by Lockhart and Martinelli^32^ and the dimensionless form suggested by Chisholm^33^. These intelligent approaches are utilized to estimate the Chisholm parameter in Eq. (8). However, before developing the new models, it is crucial to identify the most important input factors. To meet this requirement, the relevancies between Chisholm parameter and 16 common dimensionless groups were measured through the Spearman's correlation coefficients^118^, and the corresponding results have been plotted in the heatmap of Fig. 3. As it is clear, the vapor and liquid-only Reynolds number, i.e., $Re_{vo}$ and $Re_{lo}$ have remarkable impacts on Chisholm parameter. However, there is no obvious correlation between liquid-phase Reynolds number, $Re_{l}$ and C. While the vapor-phase Reynolds number, $Re_{v}$ exhibits relatively high influence of Chisholm parameter, its correlations with $Re_{vo}$ and $Re_{lo}$ are extremely great. A similar argument can be made for the liquid and vapor-phases friction factors, $f_{l}$ and $f_{v}$, which have significant correlations with $Re_{vo}$ and $Re_{lo}$. Almost all foregone experimental investigations have demonstrated that the vapor quality, $x$, and reduced pressure, $P_{red}$, play substantial roles in controlling the condensation FPD. This fact is also affirmed by the Spearman's correlation coefficient. Therefore, these factors should be considered as the models' inputs. Since the collected data include the FPD during condensation inside both mini/micro and conventional channels, the surface tension can noticeably affect the FPD. This is why the Bond number, $Bo$ shows a fairly high correlation with Chisholm parameter. The Suratman number, $Su_{vo}$, Webber number, $We_{vo}$ and Lockhart and Martinelli parameter, $X$ also can be included among the input factors, as they make unique and evident influences on C. In contrast, the impact of liquid-phase Prandtl number on Chisholm parameter is negligible. Furthermore, since the phases relative density, $R$, and the Prandtl number of the vapor phase show strong correlations with reduced pressure, they should not be incorporated as inputs to the models.

According to the above discussions, the following eight dimensionless groups were taken into account as the optimized input parameters in order to model the condensation FPD in mini/micro and conventional channels,

25 $$C = f\left( {Re_{lo} , Re_{vo} , x,P_{red} ,Bo, We_{vo} , X, Su_{vo} } \right)$$

The newly proposed models are applicable within a wide range of conditions, as shown by the minimum and maximum values of dimensionless groups in Table 3. This broad applicability reflects the extensive data bank used in this study. Since machine learning models are often sensitive to input conditions, these models offer reliable predictions for condensation FPD within the ranges summarized in Table 3.

**Table 3:** Analyzed ranges of dimensionless groups included in the novel models.

Dimensionless factor	Minimum value	Maximum value
$Re_{lo}$	327.84	232,506.9
$Re_{vo}$	1984.42	1,089,325
$x$	0.01	0.994
$P_{red}$	0.033	0.952
$Bo$	0.03	2732.708
$We_{vo}$	5.09	175,880.70
$X$	0.01	19.84
$Su_{vo}$	33,022.37	34,485,016

#### Development of the novel models

**Figure 4:**
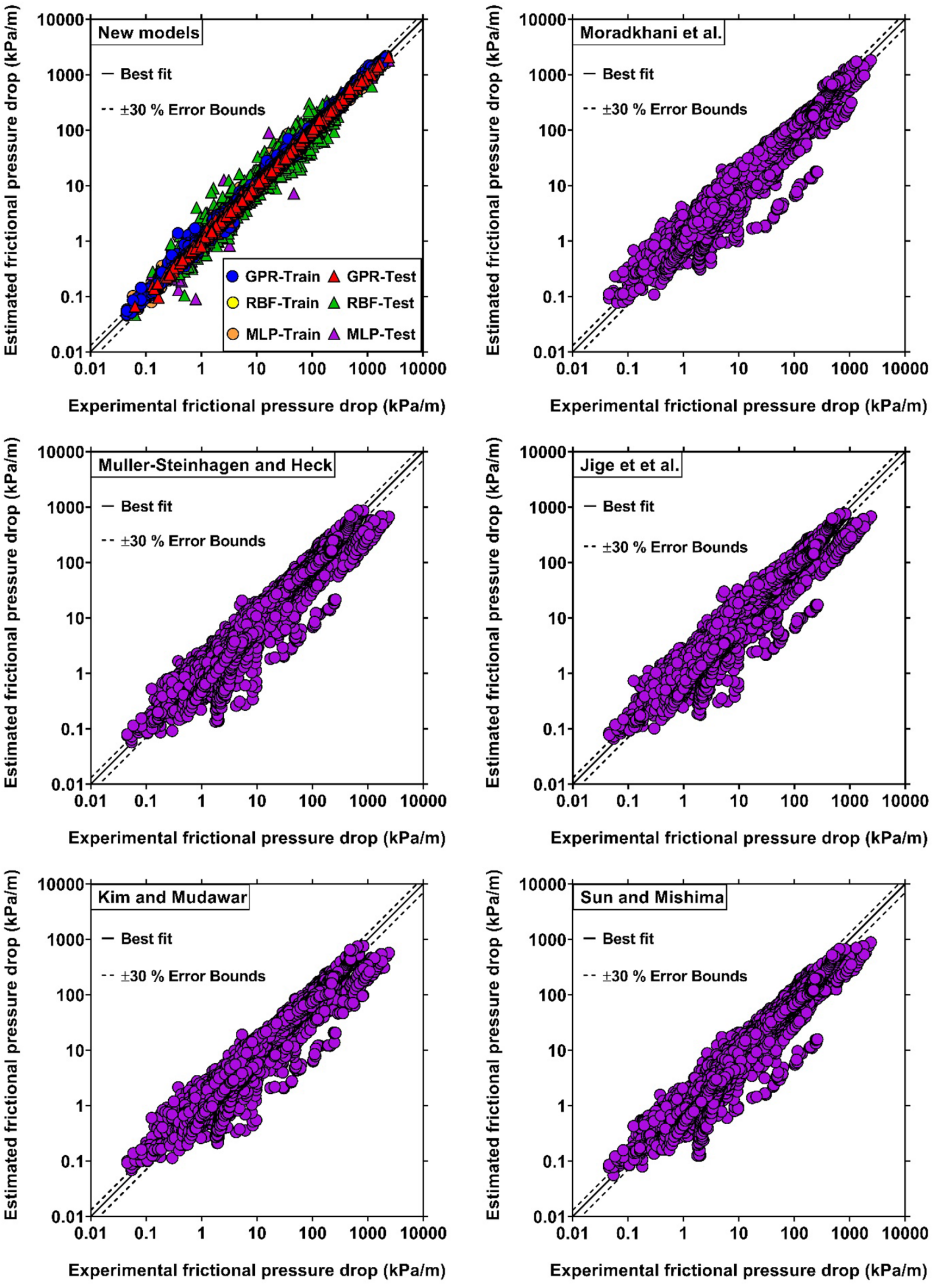
Comparison between the FPD values obtained by experimental studies and those predicted by the novel and literature models.

After determining the input variables, the soft computing approaches of GPR, MLP and RBF were enforced to derive the novel dimensionless models for predicting the condensation FPD based on the Chisholm method. This study utilized the Model-Based Calibration 5.2.1 toolbox within MATLAB to achieve this objective. This toolbox provided the necessary functionalities for designing the predictive models. Notably, the predictive tools were first trained on 80% (6,430 samples) of the randomly chosen data bank. The remaining 20% (1,607 samples) were then used to rigorously evaluate the models' reliability. The precisions of the proposed smart models during the training and testing stages have been competitively assessed in Table 4. As can be seen, the GPR-based model presents much exact estimations with AAREs of 3.77% and 4.10%, $R^{2}$ values of 99.01% and 99.23%, and SD values of 7.25% and 6.04% for train and test data, respectively. Such excellent outcomes corroborate the strength and truthfulness of this approach to describe the condensation FPD in mini/micro and conventional channels. Furthermore, it can be concluded that the optimized input variables introduced in Eq. (25) have been appropriately chosen and satisfy the influences of various factors on the condensation FPD. The MLP model also provide satisfactory outcomes, and ranks second in terms of accuracy with AARE and $R^{2}$ values of 12.46% and 97.56%, respectively in the testing phase. In addition, its predictions capabilities are adequately better those that of the literature correlations. Despite giving the best precision in the training step, the RBF model exhibits fairly great deviations for the test data points with an AARE of 21.04%. Consequently, this model cannot be assumed as a capable predictive tool. Overall, although all newly proposed models perform much better than the literature correlations in describing the condensation FPD, the one designed based on the GPR method is the best choice for this purpose. This can be justified by the fact that the GPR approach is widely recognized as a non-parametric regression tool. Unlike methods that require a pre-defined form for the data, GPR builds the model's form based on information extracted from the training samples. This flexibility allows the GPR approach to adapt to the unique characteristics and complexities of the data, resulting in improved predictive accuracy. The performance of the novel models has been compared with the top earlier correlations in Fig. 4, by plotting the estimated values of FPD versus the experimental data. The figure reflects the fact that the outcomes of the intelligent approaches, especially GPR, are impressively closer to the diagonal (best fit) line, affirming the obvious superiority of this model over the literature correlations. Among the literature correlation, the results obtained by the correlation proposed by Moradkhani et al^40^. have the best consistencies with experimental values of FPD. The rest of the correlations have almost same predictions capabilities, and this fact was also asserted by the statistical assessments provided in Table 2. Altogether, a great number of data estimated by the previous models lie beyond the satisfactory range ($\pm 30$ error margin), and the proposed model provide considerable advances in this area.

**Table 4:** Error metrics corresponding to the novel predictive tools for the condensation FPD.

	Train, (6430 data)			Test, (1607 data)			Total, (8037 data)		
Statistical parameters	GPR	MLP	RBF	GPR	MLP	RBF	GPR	MLP	RBF
AARE (%)	3.77	6.70	0.03	4.10	12.46	21.04	3.83	7.86	4.23
$R^{2}$(%)	99.01	97.60	100	99.23	97.56	95.15	99.05	97.59	99.17
SD (%)	7.25	10.50	0.39	6.04	78.12	39.00	7.74	36.53	17.70

### Assessing the validity of the newly proposed models

To further authenticate the truthfulness of the models proposed in the present study for estimating the condensations FPD inside channels, two visual accuracy analyses are carried out in this section by employing the cumulative frequency and contour diagrams.

**Figure 5:**
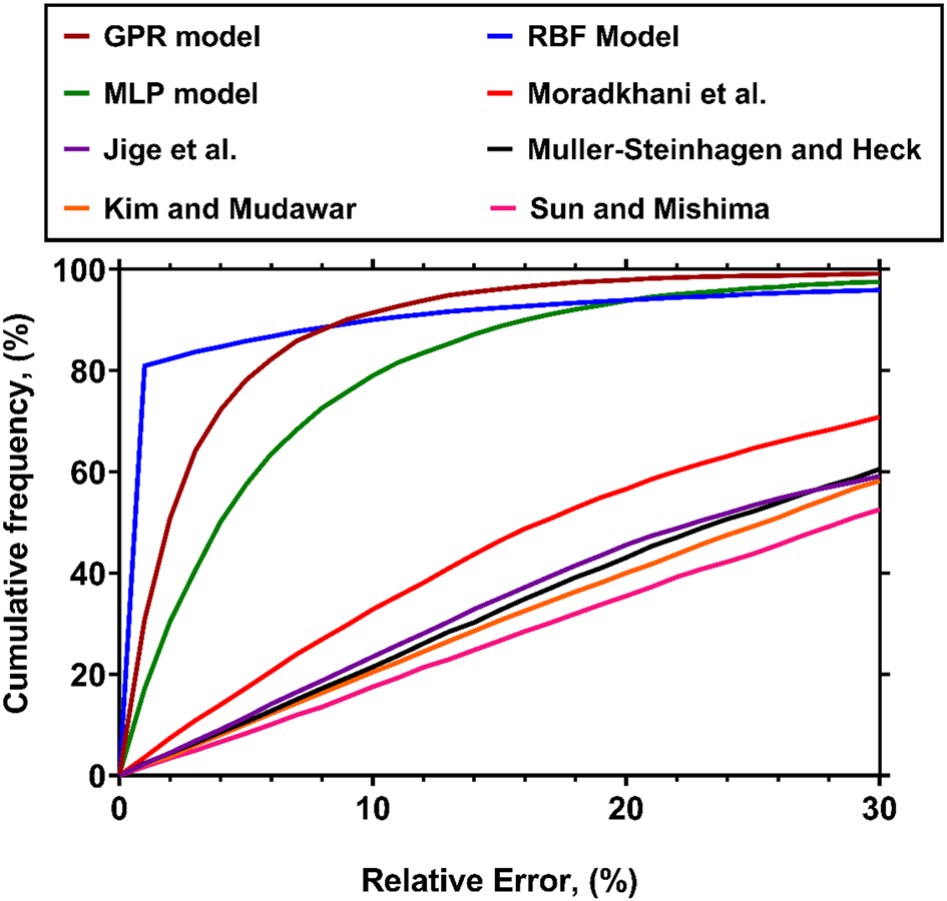
The cumulative frequency of data predicted by different FPD models at various levels of relative error.

A comparison between the reliability of the predictive approaches designed in the current study and the top models among those presented in the previous studies from the perspective of cumulative frequency has been rendered in Fig. 5. It should be expressed that the cumulative frequency measures the percentage of samples, which have been predicted within a given extent of relative error. Thus, a sharp growth in the cumulative frequency curve of a predictive tool at low levels of relative error represents its high accuracy. This observation is true regarding the novel intelligent models, since they show very high cumulative frequencies at the beginning points of its curve. Among them, the GPR approach estimates 50.78%, 78.11%, 91.45%, 96.11% and 97.92% of the whole data within the error bounds of 2%, 5%, 10%, 15% and 20%, respectively. This is why it can be recognized as the most trustful predictive tools for the condensation FPD inside channels. While the remaining intelligent models also present better results compared to literature correlations, their precisions are fairly less than the GPR model. The correlation suggested by Moradkhani et al^40^. has the best performance in terms of cumulative frequency among those available in the literature, and it is the only case that predicts the majority of samples (56.59% of all data) with relative errors less than 20%. The cumulative frequencies of the Jige et al^36^., Muller-Steinhagen and Heck^38^, Kim and Mudawar^39^ and Sun and Mishima^34^ models at the absolute relative error of 20% are 45.56%, 43.06%, 40.00% and 35.49%, respectively, which means that applying them to estimate the FPD is followed by relatively high deviations. As a results, the present analysis is another verification regarding the supremacy of the GPR model for describing the condensation FPD.

**Figure 6:**
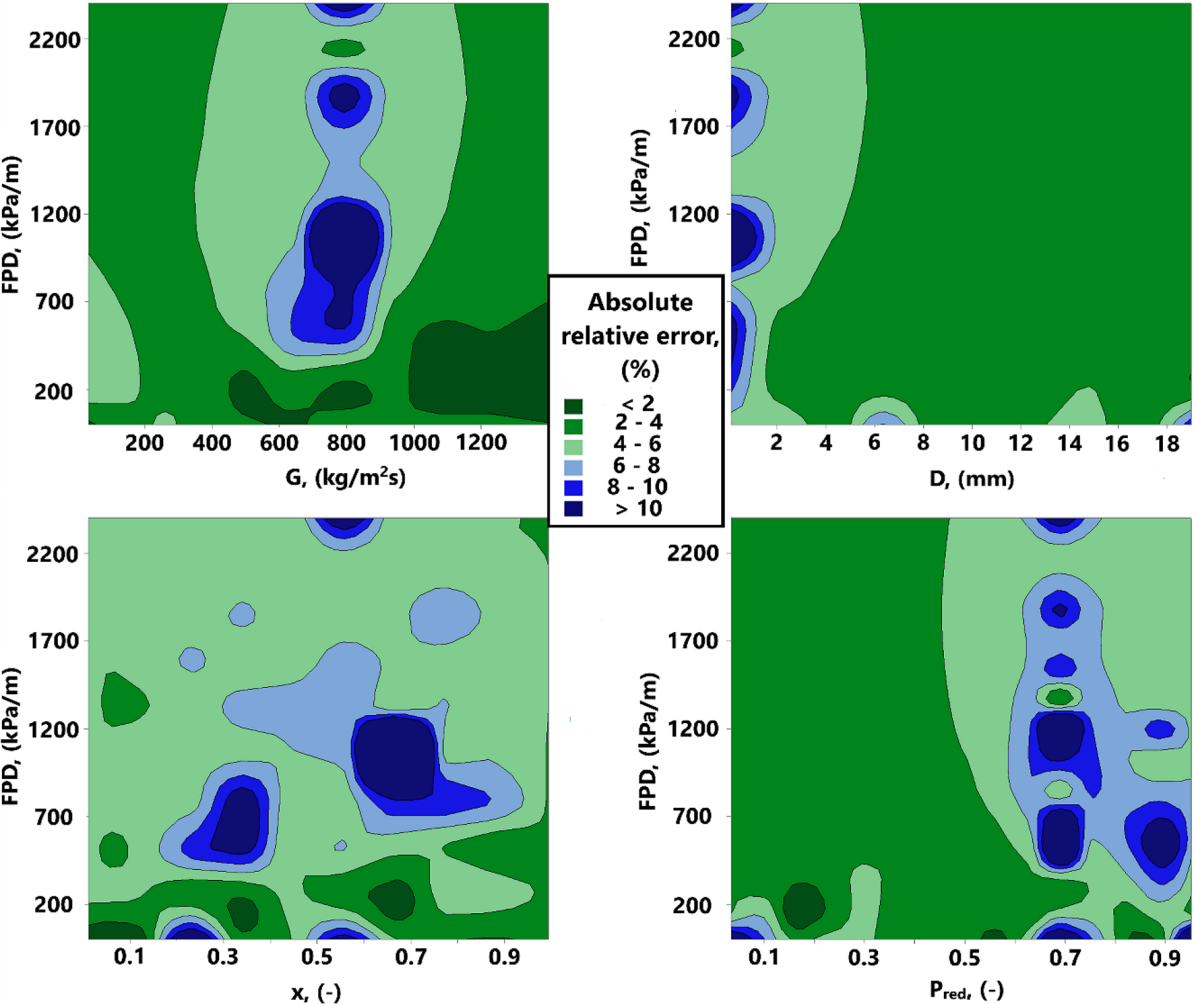
Dispersion of relative deviations given by the GPR approach for estimating the condensation FPD in diverse ranges of conditions.

The dispersion of the relative deviations yielded by the GPR approach for describing the condensation FPD in diverse ranges of flow mass flux, channel diameter, vapor quality and reduced pressure has been represented in the contour plot of Fig. 6. The spectrum of colors differing between dark green and dark blue expresses the AARE values obtained by the model in a given range of condition. As it is obvious, the predominant portion of all diagrams have been encompassed by green colors, implying the fact that the majority of FPD data in various ranges of conditions have been estimated with relative errors up to 6%. On the other hand, several small blue regions are visible in the contour plots, which are generally corresponding to the condensation of near-critical fluids with mass fluxes of around 800 kg.m^−2^.s^−1^ inside small-sized channels. Some of these minor deviations arise not only from the performance of the model, but also from the errors occurring in the FPD experimental measurements under the foregoing situations. Overall, the current results reflect the fact that the GPR model benefits from very high confidence levels in predicting the FPD during condensation inside channels.

### The prediction capabilities of the models

In order to better figure out the universality and sufficiency of the presented models, in this section, the precision of the new models to estimate the condensation FPD inside channels of different sizes as well as under various flow patterns and flow regimes is investigated, and the results are compared with the top literature correlation. Furthermore, the variations of condensation FPD versus the operating parameters are studied based on the outcomes of the model.

#### Predicting the condensation FPD inside channels of various sizes

Several criteria have been proposed to classify the channels of heat exchangers based on their size. The most prevalent one is that obtained by Kandlikar^119,120^, in which the channels can be splitted into three main categories:

- Micro-channels with $D \le 0.2$ mm
- Mini-channels with $0.2 < D \le 3$ mm
- Conventional channels with $D > 3$ mm

**Figure 7:**
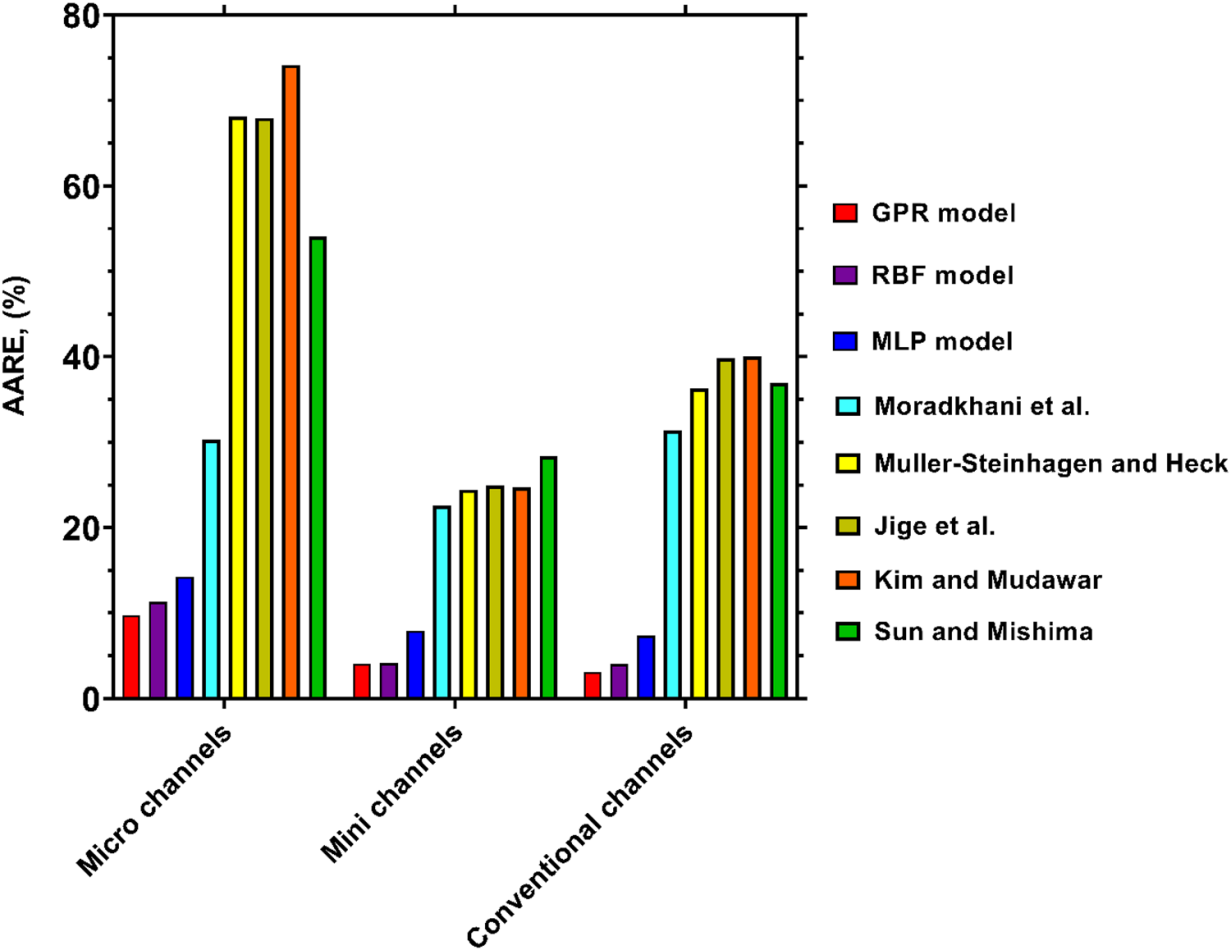
Accuracy of the novel and literature models for predicting the condensation FPD inside channels of various sizes.

By applying the foregoing classifications, the AAREs of the FPD models for the data corresponding to various sizes of channels have been illustrated in Fig. 7. The superiority of the GPR for all three cases, including micro, mini and conventional channels is obvious with AARE values of 9.74%, 4.09% and 3.13%, respectively. Such excellent results admit the applicability and reliability of this model to predict the condensation FPD in a broad range of channel sizes. The RBF and MLP models are ranked second and third from the standpoint of accuracy, since they AARE values for various channel sizes are less than 15%, and they can be used as proper alternatives to GPR approach. In contrast, the literature correlations exhibit fairly larger deviations for all types of channels, and this fact is more evident in the cases of micro and conventional channels, where all of them give AARE values exceeding 30%. Nevertheless, all of them provide relatively proper estimations for the condensation FPD inside mini-channels with AARE values of 22.56%, 24.44%, 24.69%, 24.69% and 28.35 obtained by Moradkhani et al^40^., Muller-Steinhagen and Heck^38^, Kim and Mudawar^39^, Jige et al^36^. and Sun and Mishima^34^ correlations, respectively. Accordingly, it can be concluded that the newly proposed models, especially GPR, are unique predictive tools with applicability for all sizes of channels.

#### Applicability for various flow patterns

**Figure 8:**
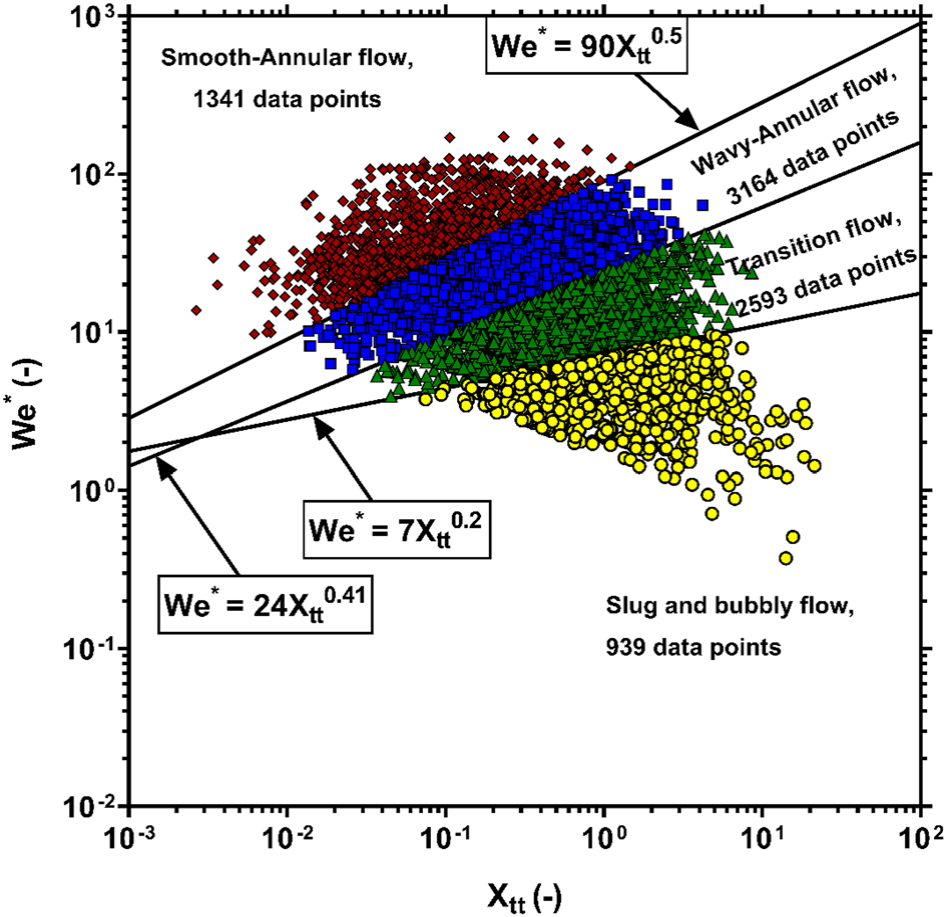
Dispersion of the analyzed FPD data in different flow patterns.

Another perceptible way to make a fair judgement regarding the performance of the models is to ascertain their accuracy level for estimating the condensation FPD at various flow patterns. Herein, the foregoing scrutiny was performed by inspiring the method proposed by Kim et al^121^., according to which four diverse flow patterns may be observed during condensation within channels. These flow patterns are distinguished from each other by three boundary lines defined based on the values of the Lockhart and Martinelli parameter, $X_{tt}$ and the modified Weber number, $W^{*}$. Figure 8 depicts the dispersion of the FPD data at all four flow patterns by determining the relevant boundary lines. As it is evident, although the wavy-annular and transition flows embody the majority of data, there are adequate numbers of samples at all flow patterns to make a comprehensive assessment on the accuracy of the models.

**Figure 9:**
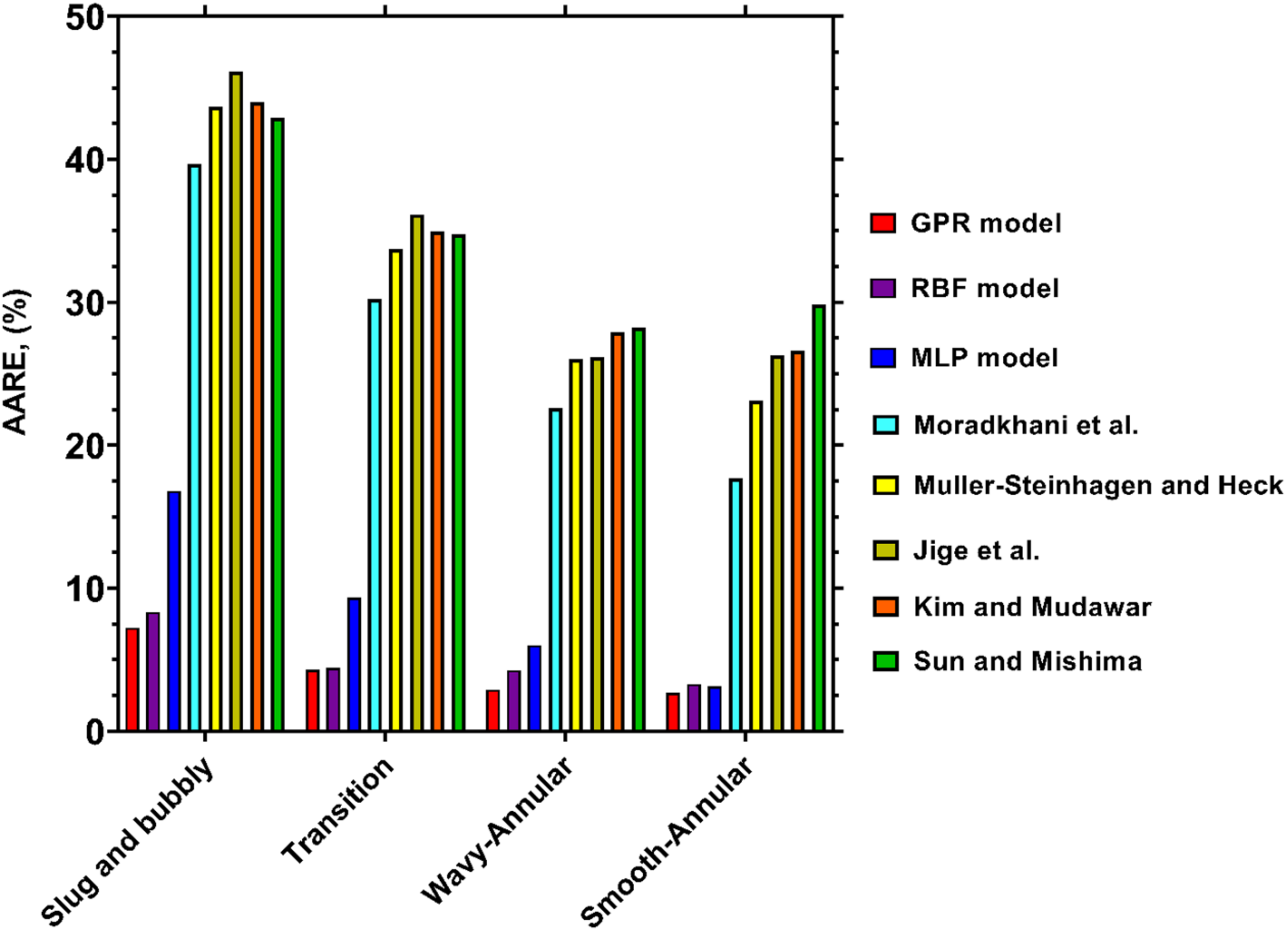
Accuracy of the novel and literature models for predicting the condensation FPD at various flow patterns.

Figure 9 portrays the AARE values yielded by the novel and earlier models for estimating the experimental data pertinent to different flow patterns. The figure clearly expresses that the highest levels of accuracy for all cases are belonged to the recently proposed GPR model. It should be noted that the AARE values obtained by this model range from 2.67% to 7.23%, which corroborates its truthfulness and potential for estimating the condensation FPD estimation in various flow patterns. The RBF model also exhibits good consistencies with the data for all flow patterns, and has AARE values below 10% for all cases. While the performance of the MLP model is superior to the literature models, the uncertainties of this model, especially for slug and bubbly flow, are higher than the other intelligent models. The predictions performed by the literature correlations are fraught with deviations, especially when the flow pattern is slug and bubbly or transitions. However, for the remaining two cases, i.e., wavy and smooth annular flows, their outcomes have slightly better consistencies with the experimental data, and the corresponding AARE values are below 30%. The most accurate predictions in the foregoing flow patterns have been presented by the Moradkhani et al^40^., correlation with AAREs of 22.59% and 17.68%, respectively. Hence, employing the novel models, especially GPR, results in noticeable improvements in predicting the condensation FPD at all flow patterns.

#### Applicability for various flow regimes

Several benchmarks have been proposed to diagnose the flow regime during condensation in channels^122–124^. Some methods consider the values of single-phase Reynolds numbers, i.e., $Re_{l}$ and $Re_{v}$ as the basis of regime classification. While, in the rest of approaches, the flow regime is specified based on the liquid-film Reynolds number, which is defined as follow,

20 $$Re_{lF} = \frac{{GD\left( {1 - x} \right)\left( {1 - e} \right)}}{{\mu_{l} }} = Re_{l} \left( {1 - e} \right)$$

where $e$ stands for the entrained liquid fraction that can be calculated by the empirical correlation developed by Cioncolini and Thome^125^. Based on the above definition, Cioncolini et al^126^. presented the following criteria for distinguishing between laminar, transitional and turbulent flow regimes,

- $$Re_{lF} \le 160$$: Laminar flow
- $$160 < Re_{lF} < 2785$$: Transitional flow
- $$Re_{lF} \ge 2785$$: Turbulent flow

**Figure 10:**
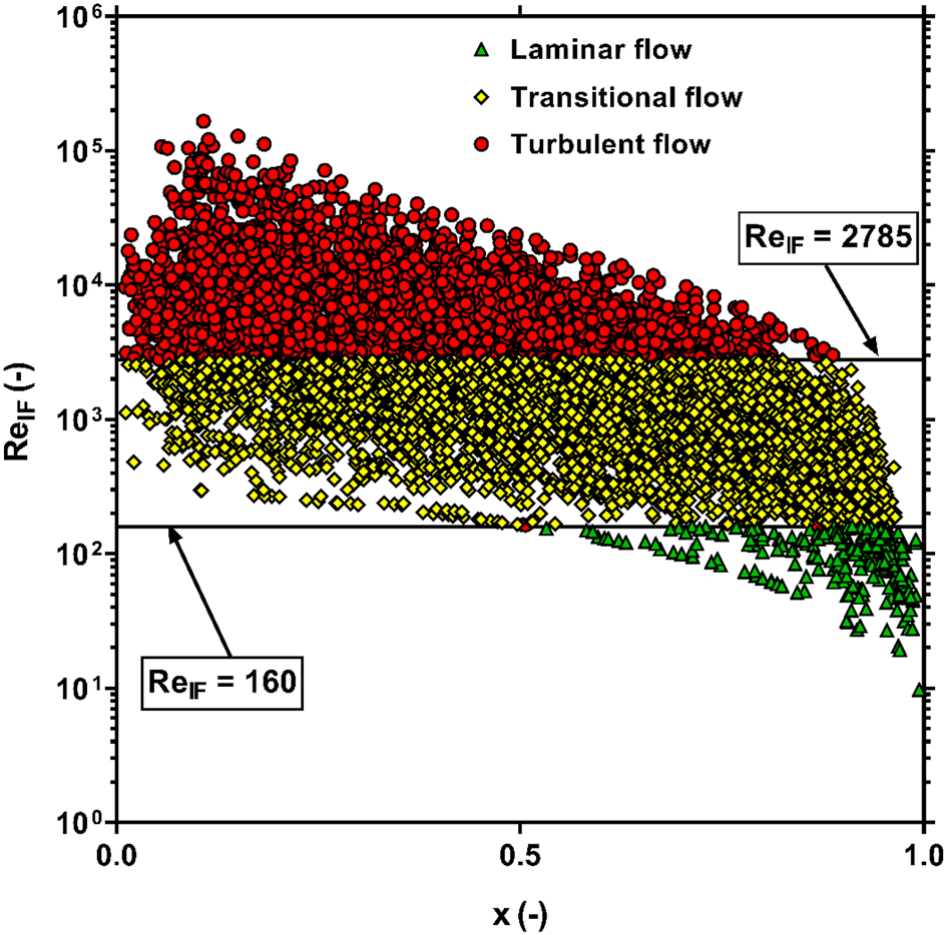
The scattering of the FPD data in various flow regimes.

Figure 10 demonstrates the scattering of the collected data at different flow regimes based on the aforementioned criteria. It is clear that the bulk of the FPD data points are included in the transitional and turbulent regimes, while the laminar regime embodies just around 3% of the entire data.

**Figure 11:**
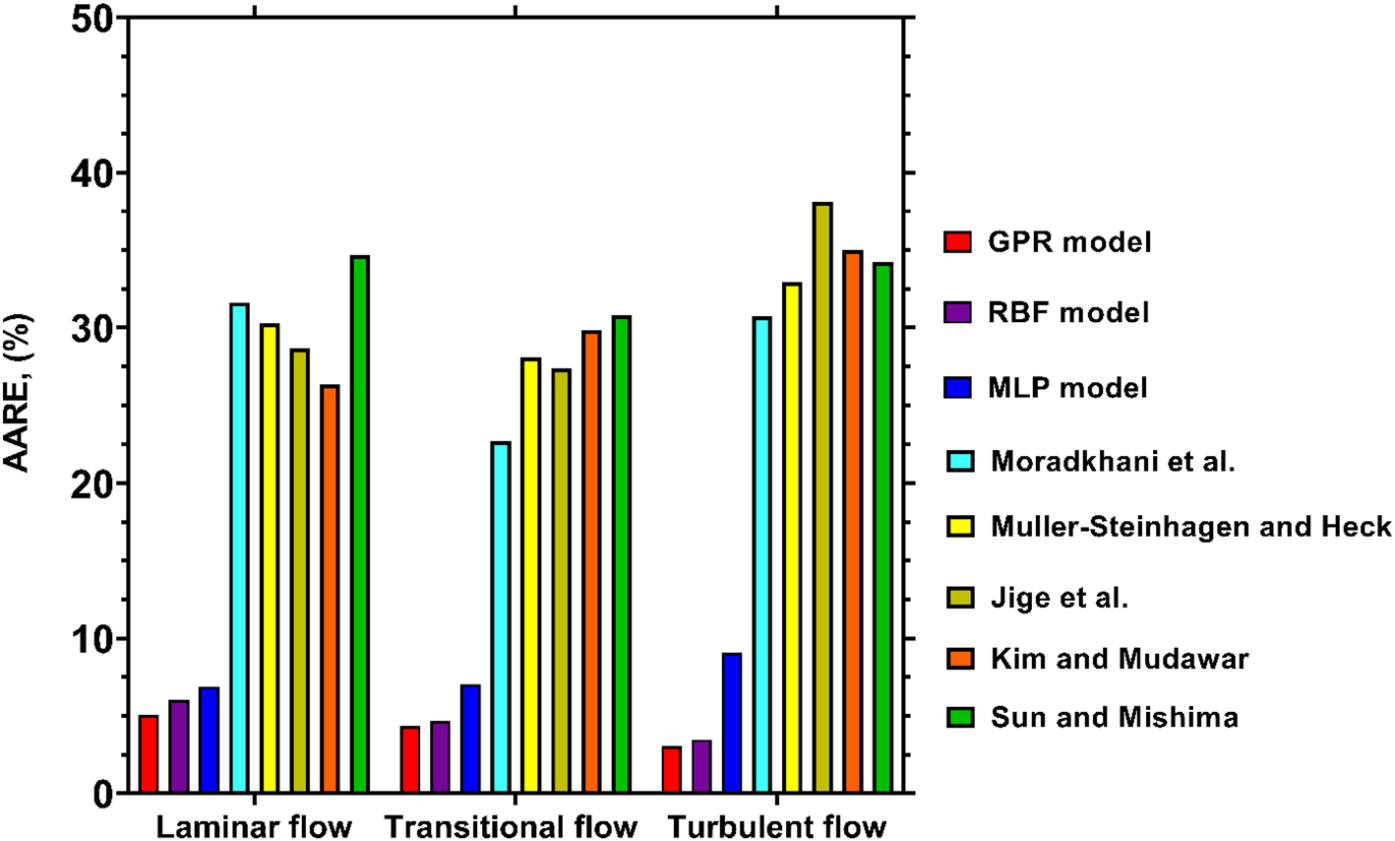
Accuracy of the novel and literature models for predicting the condensation FPD at various flow regimes.

Figure 11 compares the AARE values of different model in estimating the condensation FPD at different flow regimes. A glance at the results brings into view the decisive superiority of the GPR model over the literature correlations. The AARE values achieved by this predictive tool for various flow regimes are in the range of 3.06% and 5.06%, which is another confirmation of its universality and wide applicability. The RBF and MLP models are the next reliable predictive tool in this regard with AARE values below 10% for different flow regimes. Accordingly, the recent models provide enough accurate estimations at all two-phase flow regimes. As opposed to these approaches, the values calculated by the literature correlations are associated with relatively great errors, particularly for the turbulent flow. Although the Moradkhani et al^40^. correlation provides satisfactory outcomes for transitional flow, its AARE values for the other cases exceed 30%. It is worthy to note that with AARE values below 30%, the predictions of the models proposed by Jige et al^36^., Muller-Steinhagen and Heck^38^, and Kim and Mudawar^39^ satisfactorily match the experimental data pertinent to transitional flow. The same explanation applies to the predictions of Jige et al^36^. for the transitional flow. Thereupon, the foregoing correlations can be regarded as substitutes for the novel model in the corresponding flow regimes. Overall, except for the recently proposed models, none of the existing predictive tools are capable to precisely describe the condensation FPD in all flow regimes.

#### Capturing the physical attitudes of the condensation FPD

For exploring the potency of the recently established model, i.e., GPR to capture the physical trends, in the following, its outcomes are employed to study the variations of the condensation FPD versus the operating parameters. In order to further highlight the excellence this approach over the literature correlations, the predictions of the Moradkhani et al^40^. correlation are also included in the foregoing assessments.

**Figure 12:**
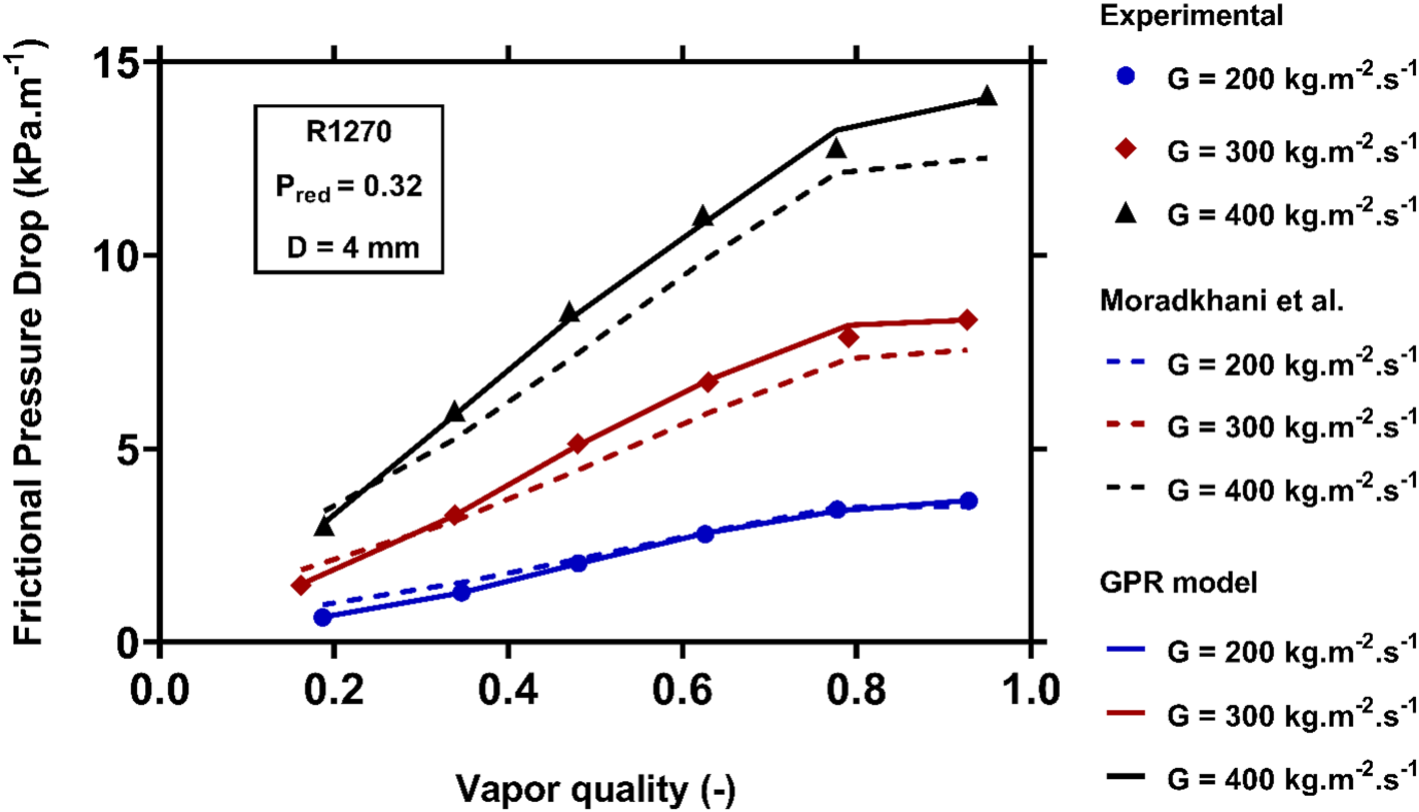
Examining the capability of the models to capture the variations of the condensation FPD versus vapor quality and mass flux.

Figure 12 delineates the variations of the condensation FPD with vapor quality and mass flux, during R1270 flowing within a 4 mm tube, when the reduced pressure is held constant at of 0.32. Obviously, the growth of vapor quality and mass flux lead to notable enhancements in the condensation FPD. This trend can be justified by amplifying the vapor velocity and shear stress arisen from the foregoing changes. While both predictive tools properly capture the overall trends, the recently proposed model are in closer conformities with the real data.

**Figure 13:**
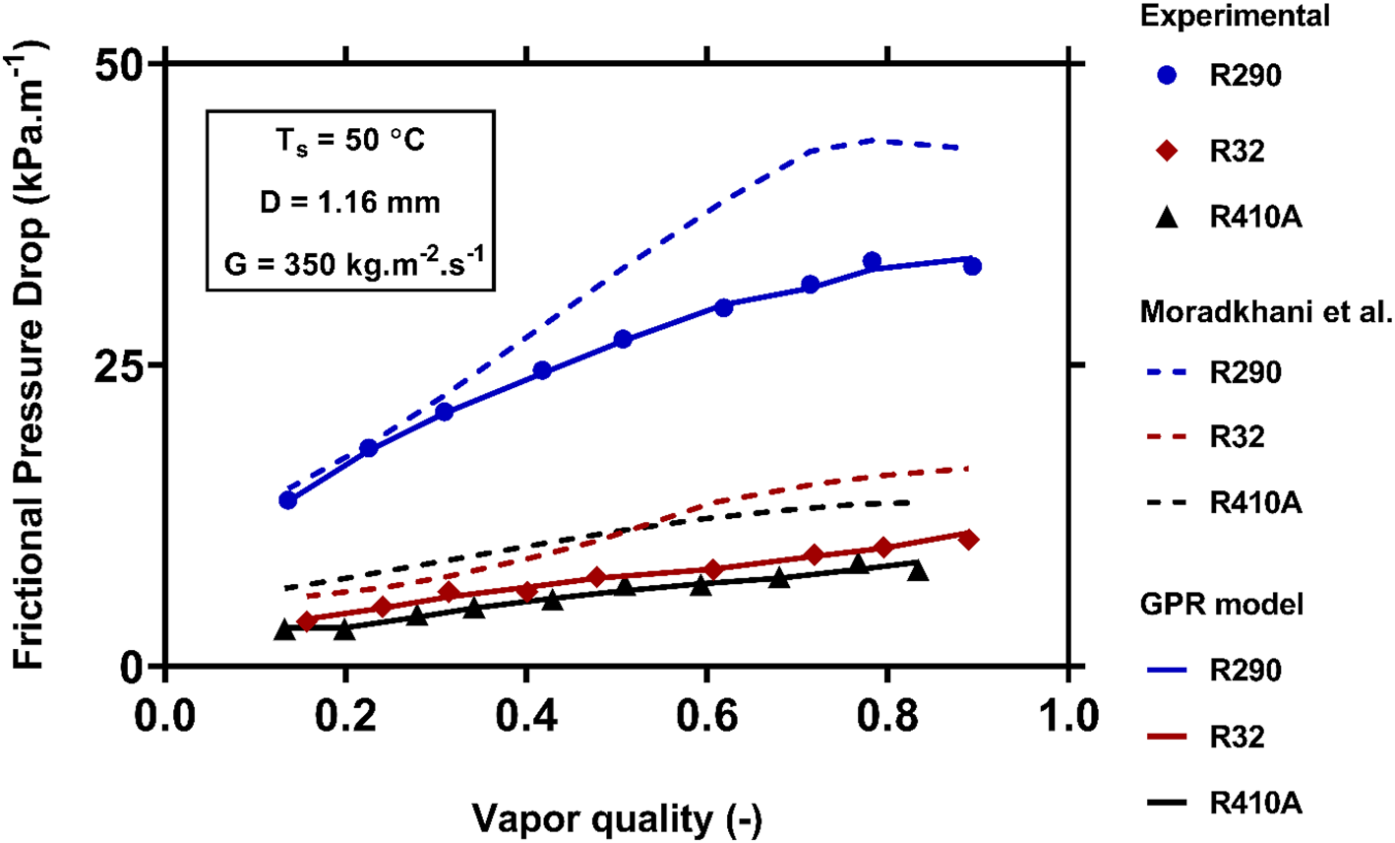
Examining the capability of the models to capture the FPD during condensation of various fluids.

Figure 13 compares the FPD variations of R290, R32 and R410A during condensation inside a channel of 1.16 mm inner diameter. As it is evident, R290 has the greatest condensation FPD among the evaluated working fluids, which is followed by R32 and R410A, respectively. Since the experiments have been carried out under constant mass flux and saturation temperature, the difference between the FPD values can be attributed to the nature of fluids. The physical characteristics of the refrigerants at the saturation temperature of 50 °C have been summarized in Table 5. As shown in the table, R290 has the lowest liquid and vapor densities, so it flows with higher liquid and vapor velocities inside the channel. On the other hand, the vapor and liquid kinematic viscosities of R290 are noticeably higher the remaining fluids. These are the reasons why highest shear stress and FPD values are observed during R290 condensation. A similar argument can be presented to justify the higher condensation FPD of R32 compared to R410A. The novel model present correct physical trends, and perfectly matches the actual data. While, the Moradkhani et al^40^. correlation over-predicts the corresponding values.

**Table 5:** Physical characteristics of R290, R32 and R410A at the saturation temperature of 50 $^\circ {\text{C}}$.

Fluids	$T_{s} \left( {{^\circ }{\text{C}}} \right)$	$\rho_{l} \left( {kg m^{ - 3} } \right)$	$\rho_{v} \left( {kg m^{ - 3} } \right)$	$\nu_{l} \left( {cm^{2} s^{ - 1} } \right)$	$\nu_{v} \left( {cm^{2} s^{ - 1} } \right)$
R290	50	448.87	38.63	0.0016501	0.0024336
R32	50	839.26	98.55	0.00099156	0.0014935
R410A	50	906.80	141.14	0.00090202	0.0014406

**Figure 14:**
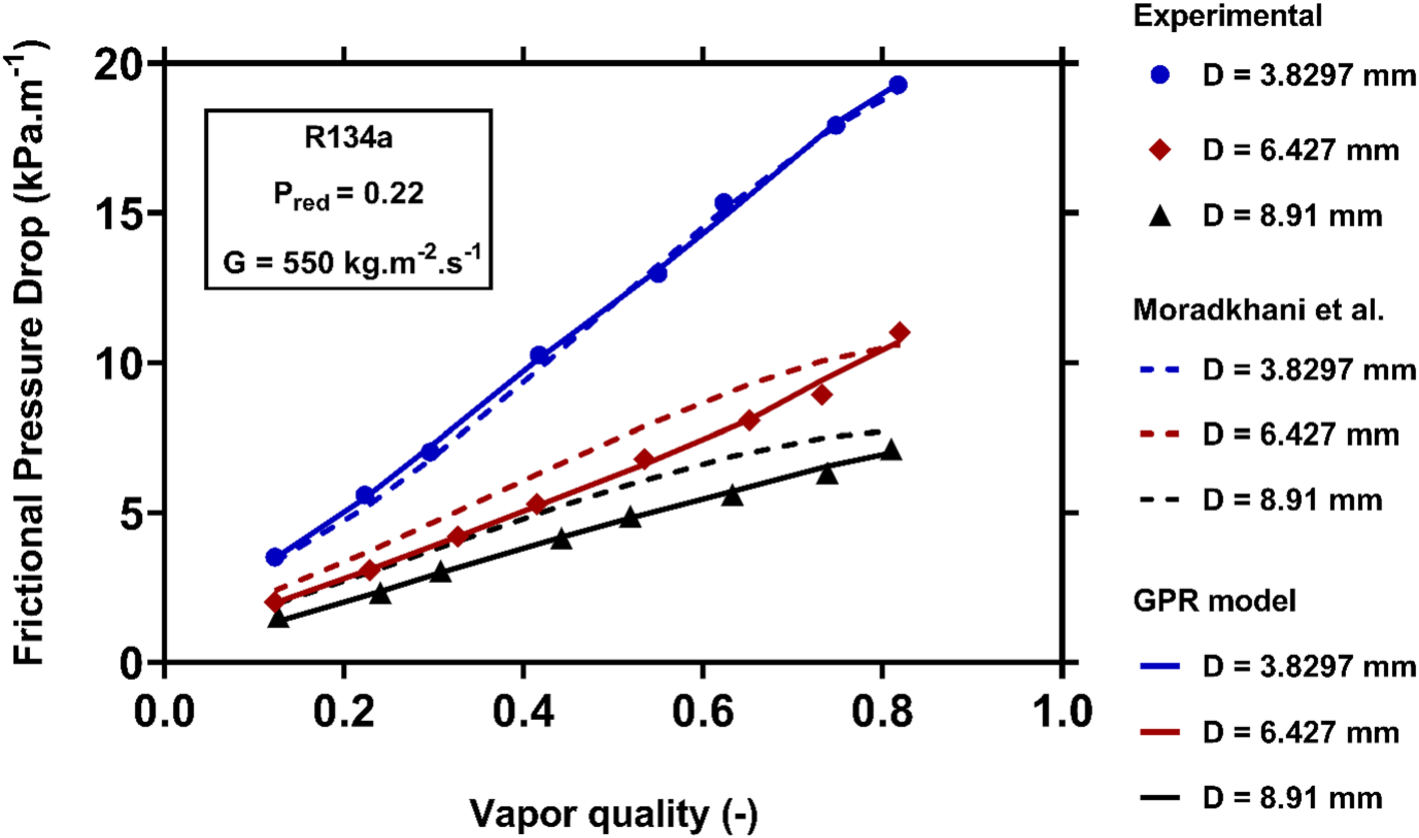
Examining the capability of the models to capture the variations of the condensation FPD versus channel diameter.

The role of channel diameter in controlling the condensation FPD of R134 under fixed operating conditions has been demonstrated in Fig. 14. The results connote the enhancement of the condensation FPD by reducing the size of channel. In fact, the impression of surface tension on two-phase flow becomes more fundamental in small-diameter channels, and can boost the velocity gradient on the wall. Consequently, the shear rate and condensation FPD are increased. Although both models present appropriate results to describe the foregoing attitudes, the predictions of the novel one have better consistencies with the measure values.

**Figure 15:**
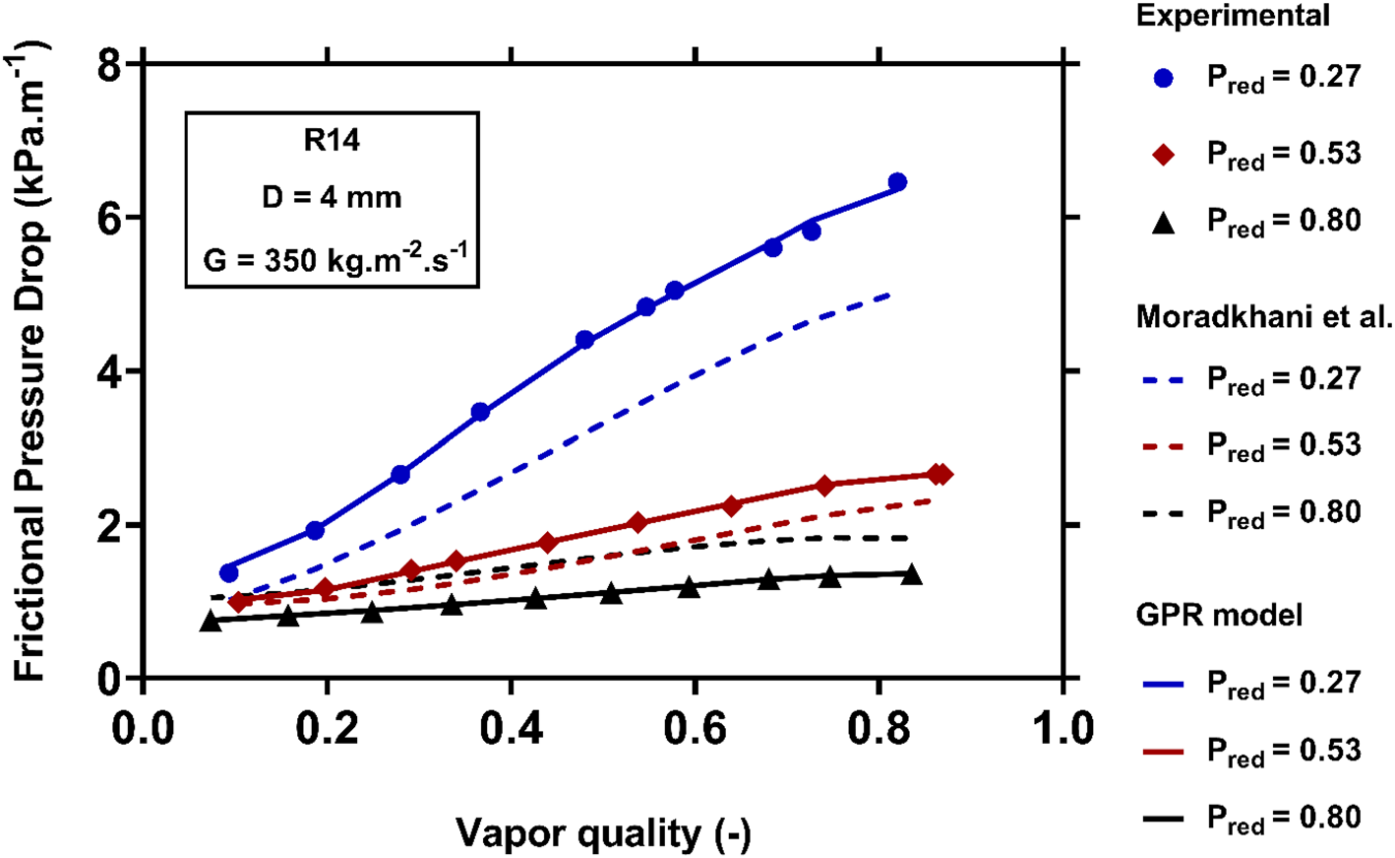
Examining the capability of the models to capture the variations of the condensation FPD versus reduced pressure.

Figure 15 sketches the alternations of the condensation FPD with the reduced pressure during R14 flowing inside a channel with an inner diameter of 4 mm at the mass flux of $350 kg.m^{ - 2} .s^{ - 1}$. A glance at the results brings it to view that the condensation FPD experiences a dramatical reduction by increasing the pressure. The principal cause of this behavior is that the phases' velocity difference at the vapor–liquid interface is amplified due to the foregoing change. While the predictions provided by the Moradkhani et al^40^. correlation have some deviations from the real samples, the GPR model excellently estimates the corresponding value.

### Sensitivity analysis

**Figure 16:**
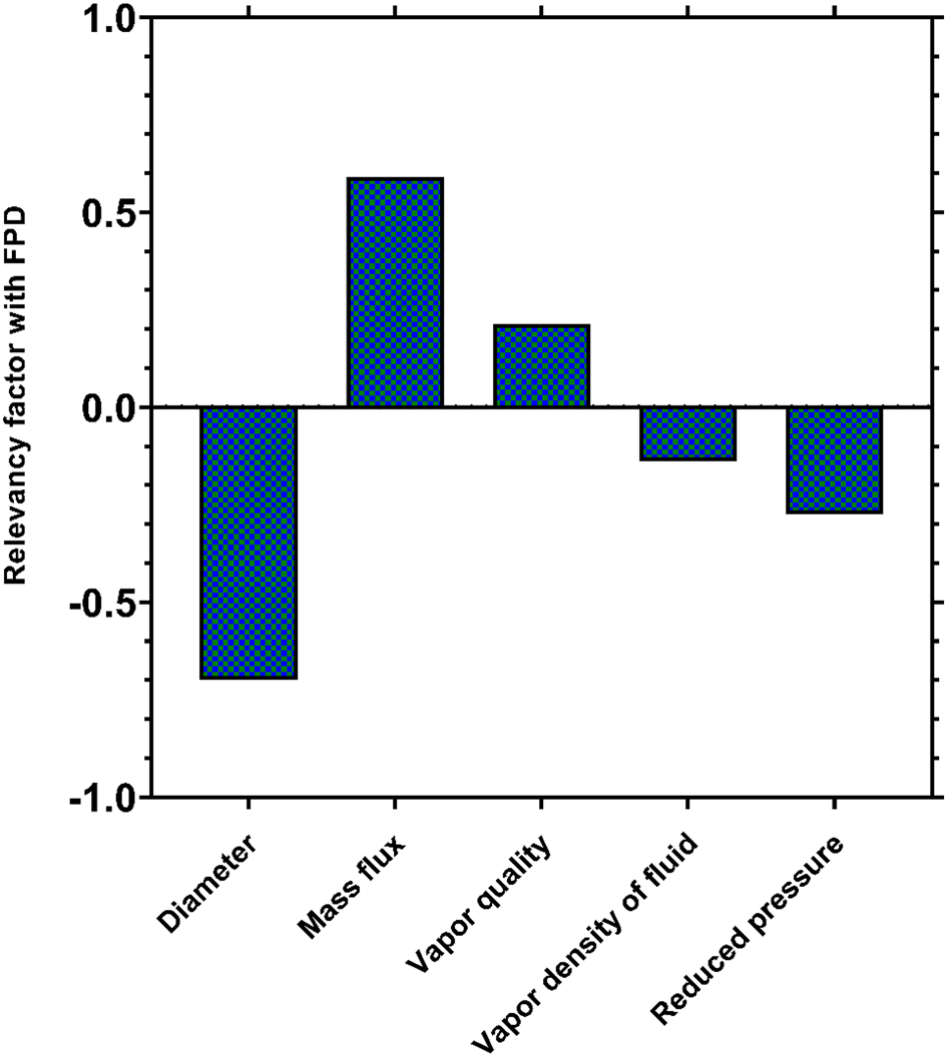
Spearman's correlation coefficient between the condensation FPD and various operating factors.

Identifying the most effective factors on the condensation FPD is another indispensable requirement that should be fulfilled for the optimal design of the heat exchangers. Accordingly, the Spearman's correlation factors between the values of the condensation FPD estimated by the GPR model and various operating factors were determined, and the corresponding results have been illustrated in Fig. 16. The figure clearly reveals that the mass flux and vapor quality have direct relationships with the condensation FPD, while the remaining factors, i.e., channel diameter, reduced pressure, and fluid vapor density inversely affect this parameter. These findings are in complete consistency with those presented in Section "The prediction capabilities of the models". Another result drawable from Fig. 13 is that the channel diameter and mass flux are the most substantial factors in controlling the condensation FPD. Furthermore, the reduced pressure, vapor quality and the vapor density of condensing fluid are ranked third to fifth from the standpoint of importance.

## Concluding remarks

This study aimed to develop robust and comprehensive predictive tools for predicting the two-phase frictional pressure drop (FPD) during condensation inside channels of various sizes. A vast dataset of 8,037 samples was collected from 50 well-regarded sources. These samples encompassed the FPD of 23 fluids, such as chemicals, halocarbons, natural refrigerants, water, hydrocarbons, cryogens, and more, over a broad range of conditions inside both mini/micro and conventional channels.

Evaluating the correctness of the literature correlations based on the gathered data denoted the point that more accurate FPD models are highly required, since all available ones gave the AARE values exceeding 26%. Hence, after choosing the optimized input variables ($Re_{lo} , Re_{vo} , x,P_{red} ,Bo, We_{vo} , X$ and $Su_{vo}$) according to Spearman's correlation analysis, the experimental data were employed for training and testing the soft computing approaches of MLP, GPR and RBF based on the theoretical method suggested by Lockhart and Martinelli^32^ and the dimensionless form suggested by Chisholm^33^.

While all the novel intelligent models outperformed the literature correlations, the GPR-based model emerged as the superior predictive tool during the testing process. It achieved remarkable performance with an AARE of 4.10% and an $R^{2}$ value of 99.23%. Additionally, the visual representations, including the cumulative frequency and contour map, confirmed that the majority of the analyzed data (over 78% of all data) were predicted with relative deviations below 5% by the aforementioned model. The MLP and RBF models also presented satisfactory results with AAREs values of 12.56% and 21.04%, respectively for the test data. The proposed models have been recognized as reliable predictive tools with excellent accuracy for estimating the condensation frictional pressure drop (FPD) inside channels of various sizes, as well as in diverse flow patterns and regimes. Furthermore, these models effectively capture the physical variations of the condensation FPD in relation to the operating factors. In contrast, the literature correlations exhibited shortcomings in most of the aforementioned analyses. A sensitivity analysis conducted using the newly developed models demonstrated that the channel diameter and mass flux play fundamental roles in controlling the condensation FPD.

In contrast to conventional correlations, which often exhibit deviations exceeding 26%, the proposed machine learning models (MLP, GPR, RBF) offer a simple, robust, and well-verified approach for FPD prediction. These models serve as accurate tools for calculating two-phase FPD during condensation in channels of various sizes. Since FPD significantly impacts the design of heat exchangers and heat pumps, the results presented here provide valuable insights for relevant engineers and designers, enabling them to optimize these systems.

For future studies, these smart approaches can be implemented to develop reliable predictive models for other crucial design parameters of heat exchangers, such as heat transfer coefficient, condensation rate, and critical heat flux. Additionally, exploring the application of deep learning algorithms for modeling two-phase flow parameters presents a promising avenue for further research.

## Data Availability

The datasets used and/or analyzed during the current study are available from the corresponding author on reasonable request.

## List of symbols

Bo: Bond number, $Bo = gD^{2} \left( {\rho _{l} - \rho _{v} } \right)/\sigma$
C: Chisholm parameter
$D$: Channel hydraulic diameter (m)
$Fr_{v}$: Vapor Froude number, $Fr_{v} = G^{2} /\rho_{v}^{2} gD$
$f$: Friction factor, defined by Eq. (7)
G: Total mass flux (kg/m^2^ s)
g: Acceleration due to gravity (m/s^2^)
k: Thermal conductivity $$({\text{W/m}}\;{\text{k}})$$
La: Laplace number
$P_{c}$: Critical pressure (Pa)
$P_{s}$: Saturated pressure (Pa)
$$P_{red}$$: Reduced pressure, $$P_{red} = P_{s} {/}P_{c}$$
$R$: Phases’ density ratio, $R = \left( {\rho _{l} - \rho _{v} } \right){\text{/}}\rho _{l}$
$Re_{l}$: Liquid Reynolds number, $Re_{l} = G\left( {1 - x} \right)D{\text{/}}\mu _{l}$
$Re_{lo}$: Liquid only Reynolds number, $Re_{lo} = GD/\mu_{l}$
$Re_{v}$: Vapor Reynolds number, $Re_{v} = GxD/\mu_{v}$
$Re_{vo}$: Vapor only Reynolds number, $Re_{vo} = GD/\mu_{v}$ (−)
$Su_{vo}$: Vapor only Suratman number, $Su_{vo} = \rho \sigma D/\mu^{2}$
$$T$$: Temperature (°C)
$$V$$: Velocity (m/s)
$We_{vo}$: Vapor only Weber number, $We = G^{2} D_{h} /\rho_{v} \sigma$
$X$: Lockhart–Martinelli parameter, defined by Eq. (9)
x: Vapor quality

## Greek

$\mu$: Dynamic viscosity (Pa s)
$$\rho$$: Density (kg/m^3^)
$\sum$: Mathematical symbol for summation
$$\sigma$$: Surface tension (N/m)
$\phi$: Two-phase multiplier
$$\upsilon$$: Kinematic viscosity (m^2^/s)

## Subscripts

*A*: Acceleration
F: Frictional
*G*: Gravitation
$$l$$: Liquid
$$lo$$: Liquid only
red: Reduced
s: Saturated
$$v$$: Vapor
$$vo$$: Vapor only
*tp*: Two-phase
